# Medical Students’ Opinions of Anatomy Teaching Resources and Their Role in Achieving Learning Outcomes

**DOI:** 10.1007/s40670-021-01436-2

**Published:** 2021-10-15

**Authors:** Elias Abdullah, Mutahira Lone, James J. Cray, Peter Dvoracek, Joy Y. Balta

**Affiliations:** 1 Department of Anatomy and Neuroscience, University College Cork, Cork, Ireland; 2 Department of Clinical Skills, School of Medicine, St. George’s University, West Indies, Grenada; 3 Division of Anatomy, Department of Biomedical Education and Anatomy, College of Medicine, The Ohio State University, OH, USA

**Keywords:** Anatomy, Education, Teaching resources, Learning outcomes, Prosections

## Abstract

Several teaching resources are used to enhance the learning of anatomy. The purpose of this study was to examine the preference of medical students on the use of various resources to learn anatomy and their link to 12 learning outcomes. A selected response item questionnaire was administered that asked students to rank six laboratory teaching resources from most to least preferred, and rate how useful these six resources were towards achieving 12 learning outcomes. These learning outcomes covered many of the learning domains such as demonstrating an understanding of anatomy, visualizing structures, appreciating clinical correlations, and understanding anatomical variations. Medical students ranked cadaveric prosections paired with an active learning clinical tutorial as the highest rank and most useful resource for learning anatomy, followed by dissection videos, electronic resources, and printed material, followed by plastinated specimens and plastic models. Overall, cadaveric prosections were also rated as the most helpful teaching resource in achieving various learning outcomes. In conclusion, anatomy teachers should provide prosections coupled with clinical tutorials as well as electronic resources as students prefer these and think they help them learn anatomy. Future studies will investigate the impact of using these resources on students’ performance.

**Supplementary Information:**

The online version contains supplementary material available at 10.1007/s40670-021-01436-2.

## Introduction

Anatomy is often referred to as the “cornerstone” of healthcare education [1, 2] and has been a crucial component of medicine throughout history [3–5]. Anatomy has also been viewed as the “first link in a long chain of events that teach new skills and competencies to tomorrow’s physicians” [6] and credited with helping to lay the foundation for safe clinical practice and communication in the field of medicine [7]. Keeping anatomy as a central part of the preclinical education has been long established as essential for the building of knowledge for both clinical education and future medical practice. To accomplish this goal, it is crucial to utilize the most effective teaching resources and methods in order to achieve the learning outcomes necessary to help shape the next generation of physicians.

When designing an anatomy curriculum, it is important to consider both the teaching methods and resources that must be integrated in order to provide an enhanced learning experience [8]. Providing a more robust sensory experience along with an active style of learning and teaching will generally lead to an effective encoding of the presented information [9]. This can include integrating additional relevant visuals, using a variety of manipulative models and specimens, or simply presenting the same information multiple times and within different contexts [10]. Some of the traditional practical teaching methods in anatomy incorporate the use of cadaveric material such as the dissection of a human body [11], teaching using prosected human material [12], and learning through plastinated human specimens [13]. Dissections, prosections, and plastinated specimens can be used to increase the level of physical manipulation and interaction, which leads to a more mentally engaged student [14]. Plastic models have also been used to teach anatomy and a recent study reported that their use aided in improving overall knowledge outcome, special knowledge acquisition, and long-term knowledge retention [15].

Other forms of practical group-based teaching have been introduced into anatomy education such as self-directed learning [16], problem-based learning [17], reciprocal peer teaching [18], peer-assisted learning, and near-peer teaching [19]. Incorporating these methods into a teaching session presents fewer technical challenges compared to using human material and, if utilized correctly, they can be a very effective methodology. Moreover, these methods can provide greater cognitive congruence between learner and teacher [19–21].

The recent advancement in technology has had an impact on anatomy education and the available teaching resources [22]. Some of these emerging technology-based teaching resources include computer-assisted learning such as digital imaging and 3D modelling [23–25], video resources [26], augmented reality [27], and virtual reality [28]. The advantage of using technology-aided learning is that students can utilize those resources in their own time and at their own pace. Unlike the traditional resources which are only available within the anatomy laboratory, technology-based teaching resources can be used anywhere without the need of high-end facilities. In addition, these resources can be personalized to the individual learner and tutorials can be attached to give students the extra guidance they need to take the next step in learning [29]. Resources like computer-assisted learning and digital imaging and 3D modelling can also be utilized in the anatomy laboratory to provide a complementary tool to the traditional teaching methods and give an extra perspective that can be useful in learning new material [30].

A mix of these more traditional and more modern teaching resources can be used in personalized combinations when providing classroom resources for the students. However, these available resources must be utilized efficiently in order to achieve the learning outcomes. With this plethora of teaching resources that can be utilized and implemented by instructors, it is necessary to use them in an effective learning combination. A recent study by Balta and colleagues provided a framework to assist faculty in utilizing teaching resources in an optimal way to achieve student learning [9]. This study suggests the Universal Design for Learning (UDL) framework as an effective tool to enhance student learning. It highlights the importance of multiple means of representation, engagement, and expression. One way to examine the effectiveness of these teaching resources is to examine the students’ perceptions of the effectiveness of these teaching resources [30–32].

Several studies have surveyed the students’ preference on the available anatomy teaching methods within different contexts [16, 30, 32, 33, 38]. For example, a study reported medical students’ ranking of resources for anatomy self-directed learning where textbooks and atlases were ranked higher than videos, software, and websites [16]. A more recent study evaluated the use of peer teaching when dissecting human material. In this study, peer teaching was perceived as an effective method of learning anatomy by more than half of the participants as peer teachers created a positive, non-intimidating learning environment [38]. Tayyem et al. reported that 49% of their students favored anatomy learning through pairing lectures and cadaveric dissection. Meanwhile, 39% favored a pairing of cadaveric dissection and multimedia as the best method of anatomy teaching [32].

Another approach to evaluate those teaching methods is by investigating medical students’ preferences on how well each resource or method helps them achieve specific learning outcomes. Two studies were reported in the literature comparing students’ perceptions on how anatomy teaching methods helped them achieve a list of learning [30, 33]. In the study by Kerby et al. in 2011, medical students ranked prosection and demonstration as the top teaching method when it comes to achieving anatomy learning, clinical background, medical vocabulary, 3D appreciation and appreciation to variation in comparison to models, and computer-assisted learning (CAL) [30]. These findings are similar to those reported by Chapman et al. in 2013. In this study, prosections were rated the highest by students for helping them instill anatomical knowledge, appreciate clinical anatomy, 3D appreciation, and anatomical variations [33]. This aligns with Kolb’s experiential learning framework where learners are able to create knowledge from experience [39].

In this study, we aimed to investigate medical students’ perceptions on their anatomy learning process by evaluating six teaching resources on how helpful they found them in achieving twelve learning outcomes in the context of the anatomy teaching curriculum at University College Cork. This study comes to fill the gap in the literature addressing the link between teaching resources and their effectiveness in fulfilling learning outcomes. For this reason, in this study, we assess how helpful did the students find those six teaching resources in achieving 12 learning outcomes.

## Material and Methods

### Anatomy Practicals

Graduate entry medical students at University College Cork (UCC) learn anatomy through a combination of lectures, laboratories, and problem-based learning. The gross anatomy curriculum is delivered in the first year and the neuroanatomy curriculum in the second year. The anatomy curriculum is divided into four main blocks (upper and lower limbs; cardiovascular and respiratory systems; abdomen, male/female reproductive organs, and pelvis; and neuroanatomy) with a total of 163 h (30% lectures, 70% laboratory practical sessions) [9]. In the laboratory session, students are assigned to a particular group and work in a rotation system. Each group has an average of four–six students which may be working with prosections, plastic models, plastinated specimens, histological slides, and computer-assisted learning tools (Anatomy & Physiology Revealed) including videos (Acland’s Video Atlas of Human Anatomy) and literature (books and atlases). The students rotate to a new station every 20–25 min, thus allowing them to encounter every component of the assigned laboratory work. The prosection stations are delivered by faculty members through an active learning clinical tutorial. During this time, faculty engage with the students by asking questions trying to connect the content learned in lecture to that which they examine on a prosection. All other stations are student led using peer-assisted learning model where they use a lab handbook to work together and go through the content outlined for this specific session. Students are assessed through a laboratory practical examination (spotter exam) where they have 1 min at each station with 20 stations in total. Each station has parts A and B with A usually being an identification question and part B asking more information about the identified structure.

### Study Design

In addition to lectures, the teaching of the first year medical students is supported by the university’s web-based learning tool BlackboardTM that facilitates communication between faculty and students as well as allowing students to have access to Acland’s videos and library e-books. First year medical students were invited to participate in this study and were given information about the project via Blackboard notification.

Data was collected at the end of the spring semester during the abdominal gross anatomy system after the completion of the upper and lower limbs and cardiovascular/respiratory system. On the day, students were reminded about completing the questionnaire during their practical sessions and completed forms were collected at the end of the laboratory session. An informed consent form was attached to the questionnaire which all students had to fill out before completing the questionnaire. The signed consent forms were separated from the questionnaires after collecting all the documents in order to anonymize the data.

### Ethical Approval

Personal identifiers were removed from the data set before being analyzed. The quantitative data was compiled using Microsoft Office—Excel. Ethical approval was granted for this project by the Social Research Ethics Committee (SREC) at University College Cork under log number 2018–023.

### Questionnaire Design

A total of 86 first year medical students were enrolled in the course and all the students present at the laboratory were given a questionnaire handout. A questionnaire was designed for the study which constituted of two parts: participants’ background and anatomy learning experience. Invitations were sent to the eight anatomy master’s students in the Department of Anatomy and Neuroscience in University College Cork to participate in a pre-test study. Feedback from the pre-test study was discussed in a focus group session and questions were altered based on the feedback session.

The aim of the first part was to gather more information about the participants’ backgrounds. In the second part, the students ranked laboratory teaching resources from most to least helpful, and then rated the usefulness of each resource for achieving 12 learning outcomes. The 12 learning outcomes are listed below along with their link to the three UDL principles:

*Multiple means of representation*: 3D visualization; anatomical features; anatomical variations; functional anatomy.

*Multiple means of engagement*: body systems and relationships; interrelationships; anatomical terminology.

*Multiple means of action and expression*: spatial relationships; locating anatomical structures; clinical aspects; recall of anatomical information; understanding gross anatomy.

Questions were divided into multiple choice, ranking, and rating questions. The questionnaire has been attached as supplementary material.

### Statistical Analysis

The anonymized data was collected and entered manually into Microsoft Excel spreadsheets. Data was then exported to the Statistical Package for Social Sciences (SPSS) (IBM Corp., Armonk, NY) in which the Friedman (for > two variables) and Dunn’s test (for two variables) were used to determine statistical significance when comparing two different resources.

An alpha of 0.05 was used as the cutoff for significance for the Dunn’s test. Dunn’s test was conducted with a Bonferroni correction applied and adjusted significance levels from SPSS were used.

## Results

### Demographics

A total of 86 first year medical students were enrolled in the course with 65 students taking part in the study yielding a 76% response rate. The first part consisted of four background-related questions which included gender, age, country of origin, and previous degree(s). Results of the background questions are outlined in Table 1.

**Table 1:** The age, country of origin, previous degrees, and gender of participating students

**Demographics**	**Number of students (*N*)**					
Age	20–23 (24)	24–26 (29)		27–30 (8)		Over 30 (4)
Country of origin	Ireland (23)	Canada (30)		UK (5)		Other (7)
Previous degree	Science (47)		Non-science (15)		Other (3)	
Gender	Female (37)			Male (28)		

### Preference Ranking of Various Resources

**Fig. 1:**
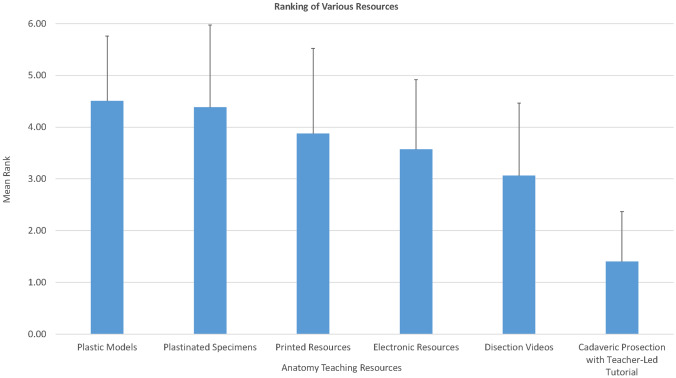
Participating students’ ranking of teaching tools with one being most preferred and six being least preferred

Study participants were asked to rank the resources from one to six with one = most preferred and six = least preferred. Cadaveric prosection which was used as part of a clinical tutorial (mean = 1.40, *SD* = 0.965) was ranked as the most preferred for learning anatomy and plastic models (mean = 4.51, *SD* = 1.252) were the least preferred learning tool. Analysis by participant demographics did not yield any significant differences in their most preferred learning resource. One exception was the age groups 27–30 and over 30 years old who chose printed and electronic resources as the least helpful aids. The results from the matrix questionnaire are represented graphically in Fig. 1 in which one is used to represent most preferred and six being least preferred.

The difference (via the Friedman’s test) between the six various resources was statistically significant (*χ*^2^ (5, *N* = 65) = 121.876, *p* < 0.001p). Dunn’s test was conducted with a Bonferroni correction applied and adjusted significance levels were used. A statistically significant difference (*p* < 0.001) was observed when comparing cadaveric prosection with all other resources along with the comparison between dissection video and both plastinated specimen/plastic model (cadaveric prosection–dissection video: *t* = 1.646, *p* < 0.001; cadaveric prosection–electronic resources: *t* = 2.192, *p* < 0.001; cadaveric prosection–printed resources: *t* = 2.469, *p* < 0.001; cadaveric prosection–plastinated specimens: *t* = 2.977, *p* < 0.001; cadaveric prosection–plastic models: *t* = 3.085, *p* < 0.001).

### Student Ratings of Usefulness of Learning Resources for Achieving Learning Outcome

**Fig. 2:**
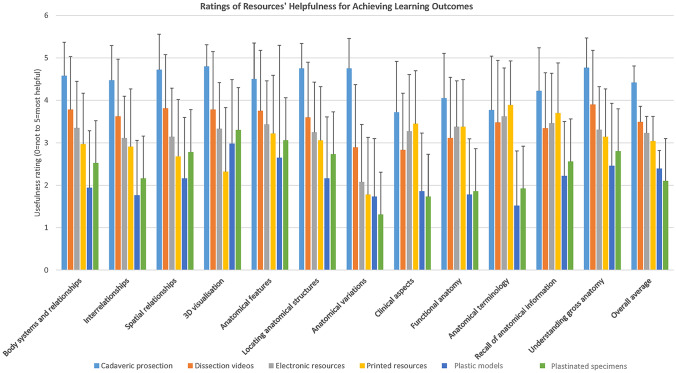
Participating students’ ratings on how useful the resources were to achieving each of the 12 learning outcomes with zero being not helpful and five being most helpful

Students were asked to rate the available resources in the anatomy laboratory on how helpful they were in achieving various learning outcomes by using a six-point scale (zero = not helpful and five = most helpful). Figure 2 shows in detail how the participating students rated cadaveric prosection, dissection videos, electronic resources, printed resources, plastinated specimens, and plastic models as helpful tools to achieve the 12 learning outcomes. Overall, cadaveric prosections were rated as the most helpful teaching resource in achieving various learning outcomes followed by videos, electronic resources, printed resources, plastic models, and plastinated specimens were considered as the least helpful. The difference (via the Friedman’s test) between the six various resources was statistically significant (*χ*^2^ (5, *N* = 65) = 138.888, *p* < 0.001). Dunn’s test was conducted with a Bonferroni correction applied and adjusted significance levels were used. The overall difference between all the resources was statistically significant except for plastinated plastic and printed electronic with *p* > 0.05. Plastinated specimen–dissection video: *t* = 1.927, *p* < 0.001; plastinated specimen–cadaveric prosection: *t* = 3.113, *p* < 0.001; plastic specimen–dissection video: *t* = 1.710, *p* < 0.001; plastic–cadaveric prosection: *t* = 2.895, *p* < 0.001; printed material–dissection video: *t* = 1.242, *p* = 0.003; printed material–cadaveric prosection: *t* = 2.427, *p* < 0.001; electronic material–dissection video: *t* = 1.048, *p* = 0.027; electronic material–prosection video: *t* = 2.234, *p* < 0.001; dissection video–prosection video: *t* = 1.185, *p* = 0.006. Further analysis of the sample population also showed prosection as the most preferred learning aid by all nationalities, age groups, genders, and including those with science and non-science backgrounds. It is also worth mentioning that similar learning outcomes such as interrelationships, spatial relationships, and 3D visualization received similar rating which supports the validity of the questionnaire.

## Discussion

The findings of this study show that medical student ranked cadaveric prosection when used as part of a clinical tutorial as the most preferred anatomy laboratory teaching resource. This was followed by dissection videos, electronic resources, printed resources, plastinated specimens, and the least preferred was plastic models. Overall, similar outcomes were concluded when asked to rate the available resources in the anatomy laboratory on how they helped them achieve various learning outcomes.

The anatomy lab system including the rotation of students between the different stations using different teaching aids aligns with Kolb’s experiential learning theory (ELT) which allows the students to obtain knowledge, construct it, and apply it in dynamic experiences. There are 4 elements to Kolb’s ELT which can be linked to the anatomy learning stations. During the faculty-led prosection station along with the Acland’s videos, students have a concrete experience followed by reflective observations during the self-directed stations. Moreover, students also have the opportunity for active experimentation during the practice spotter exam as part of the practical session [39].

### Student Preferred Learning Resource

By utilizing a six-point scale, the medical students ranked cadaveric prosection coupled with a clinical tutorial as the most preferred teaching aid for learning anatomy. These findings corroborate with those published by Tayyem et al. where prosection and demonstration was ranked higher than models and CAL. The main reason for prosections being highly preferred could be due to the fact that a faculty member utilized this resource as part of a clinical tutorial where students were able to appreciate the clinical relevance of the material being taught. Other reasons could be related to students’ ability to touch, feel, and have a hands-on experience on a realistic specimen in comparison to a plastic model. Moreover, students were able to appreciate spatial relationship of different anatomical structures, visualize 3D structures, anatomical variations, and identify the different anatomical features on a prosected specimen which is not something that they were able to achieve on a plastinated specimen due to the fixed nature of the tissue. It can therefore be deduced that students perceived the use of prosections in the laboratory as an effective way of learning anatomy.

Dissection videos, a type of information and communication technology, also referred to as a technology-enhanced learning tool, were the second highest ranked resource. Using videos is considered very helpful for grasping concepts and visualizing anatomical relations [34]. In comparison to our findings, videos were also rated highly by students as a tool that helped them in visualizing 3D anatomy of the structures and associate the various body system, but not as highly as cadaveric prosections. However, different studies reported contradicting outcomes on the effectiveness of using videos in anatomy teaching, with some indicating that such resources did not help in student performance [26], while another study reported that videos are helpful, easily accessible, and affordable with improved student learning activity in a limited time [34]. Plastinated specimens and plastic models were ranked as the least helpful teaching aids.

### Teaching Aids and Learning Outcomes

The students were asked to rate six teaching resources related to 12 learning outcomes. A six-point scale was used to rank the resources with the outcomes. The averages of all the means were calculated and cadaveric prosection and dissection videos were deemed the most favored resources that helped achieve the learning outcomes. The difference between cadaveric prosection and dissection videos was calculated using Dunn’s test and results showed a significant difference (*p* < 0.001).

Prosections have been viewed as useful for exploring, visualizing, and understanding interrelations of structures [35]. Results from this study are in line with those reported by Kerby et al. [31] in which students ranked the cadaveric prosection teaching aid very high in reaching five of the shared learning outcomes investigated in this study. These outcomes are related to imparting anatomical information, appreciate and provide background clinical information, obtain 3D appreciation, develop vocabulary, and appreciate anatomical variation. Relevant research by Patel and Moxham in 2008 also showed that professional anatomists also hold prosected specimens in high regard for achieving learning outcomes [36].

In contradiction to the findings of our study, plastinated specimens were found to be very useful by Latorre et al. [37]. A study at Warwick Medical School, UK, also showed that the majority of students (94%) deemed plastinated specimens as a “valuable resource” in learning anatomy [13]. In addition, students welcomed the plastinated specimens because they were able to appreciate relevant details and relationships along with visualizing the specimens in real life. Limitations were related to emotional and tactile reality [13]. One explanation for this difference could be due to the fact that students were being guided by a faculty member when studying with/being taught on the prosected specimens, whereas the plastinated specimens were used for self-directed study and hence maybe failed students failed to appreciate the relevance of these teaching aids. For each of these stations (prosections and plastinated specimens), students had 20–25 min for studying the anatomical features.

It is generally accepted that visualization is an important tool in the procurement of anatomical knowledge. A study by Lone et al. showed that animations can act as a supplemental aid in the study of cranial nerves [37]. The first year medical students that participated in this study were offered videos and CAL as a form of animation tools. The mean of all combined averages in this study of the 12 learning outcomes showed that electronic resources (CAL) received a high mid-rank position (rank = four, mean = 3.23). When comparing the five similar learning outcomes investigated in this study and that by Kerby et al. there is clear consensus that CAL is ranked second or third along with plastic models with prosections being ranked first. Meanwhile, the study by Chapman et al. shows that CAL and the plastic models were rated similarly for helping students instill anatomical knowledge, appreciate clinical anatomy, and obtain 3D appreciation of the body. The only difference between those two resources was helping the students appreciate anatomical variations where the students found CAL more helpful than plastic models. These findings are similar to those by our study and those by Kerby et al.

From this study, printed material was an exception in which it was chosen by the first year medical students as the best resource (*n* = 64, mean = 3.89) for developing a vocabulary of anatomical terminology to effectively communicate anatomical information. A valid reason for choosing printed material as a helpful source for developing a vocabulary of anatomical terminology is that books/atlases are known to be inextricably associated with authorities/knowledgeable authors. Meanwhile, printed material was rated as the least helpful (*n* = 63, mean = 2.32) for visualizing the 3D anatomy of the structures. The difference between these two learning outcomes was calculated to be statistically significant (*p* < 0.05). Printed materials are by nature 2D, although they may attempt to depict 3D structures, so it is expected that students would rate them as not helpful for visualizing 3D structures compared to prosections and other 3D models. However, the findings of this study show that printed materials are still able to provide an added value to the students’ learning process.

Although this research along with the questionnaire was carefully planned and carried out, some shortcomings and limitations were unavoidable. The study was carried out in a single institution with only 65 students taking part which is a small sample size. Moreover, there were neither any pre or post studies done nor any measured and evaluated or calculated longitudinal mechanisms that helped to appreciate any fluctuations in opinion over time. In this study, we did not evaluate actual usage and correlations with learning outcomes. This would be difficult to do as students had a standard amount of time at each station, but perhaps they did not engage as much at some stations. Another limitation that could have impacted the comparison between prosected and plastinated specimens is the fact that prosected specimens were used to run a tutorial with students while plastinated specimens were self-directed.

The findings of this study contribute to the currently existing literature by adding six learning outcomes to those investigated in previous studies along with focusing on the laboratory teaching resources. With the current push to eliminate the use of prosected human material and strictly utilize plastic models and plastinated specimen, this study comes to highlight the benefits of providing the students with human material that they can manipulate in order to achieve a list of learning outcomes. The findings of this study will also help us improve the way we use the anatomy teaching resources knowing that the students strongly prefer a more guided approach similar to that delivered using cadaveric prosections.

## Conclusion

Connecting teaching resources to the outlined learning outcomes is very important for student learning. While technology-based resources are very helpful to learn anatomy, in the opinion of students, human material remains the most superior way to teach anatomy and help achieve the biggest number of learning outcomes. Utilizing human material under the guidance of teaching staff can be an added value to the learning process. Therefore, it is important for anatomy teachers to find meaningful ways to utilize technology-based resources, while highlighting the benefits of human material in the learning process. Future studies will need to assess the benefits of using those learning resources to students’ performance.

## Supplementary Information

Below is the link to the electronic supplementary material.

Supplementary file1 (PDF 61 kb)

## References

[1] Balta JY, Cronin M, Cryan JF, O’Mahony SM (2015). Human preservation techniques in anatomy: a 21st century medical education perspective. Clin Anat.

[2] Papa V, Vaccarezza M. Teaching anatomy in the XXI century: New aspects and pitfalls. Scio World J. 2013;310348. 10.1155/2013/310348 PMC3842041 24367240

[3] McLachlan JC, Patten D (2006). Anatomy teaching: ghosts of the past, present and future. Med Educ.

[4] Shoja MM, Tubbs RS (2007). The history of anatomy in Persia. J Anat.

[5] Korf HW, Wicht H, Snipes RL, Timmermans JP, Paulsen F, Rune G, Baumgart-Vogt E (2008). The dissection course—necessary and indispensable for teaching anatomy to medical students. Ann Anat.

[6] Pawlina W, Lachman N (2004). Dissection in learning and teaching gross anatomy: rebuttal to McLachlan. Anat Rec.

[7] Turney BW (2007). Anatomy in a modern medical curriculum. Ann R Coll Surg Engl.

[8] Balta JY, O’Keeffe GW, Supple B (2019). Utilizing the scholarship of teaching and learning to design an anatomy pedagogy course Eur. J Anat.

[9] Balta JY, Supple B, O’Keeffe GW. The universal design for learning framework in anatomical sciences education. Anat Sci Edu. 2020. 10.1002/ase.1992 32539206

[10] Najjar LJ. The effects of multimedia and elaborative encoding on learning (GIT-GVU-95–29) Atlanta, GA: Georgia Institute of Technology, Graphics, Visualization and Usability Center. 1997.

[11] Snelling J, Sahai A, Ellis H (2003). Attitudes of medical and dental students to dissection. Clin Anat.

[12] Mitrousias V, Varitimidis SE, Hantes ME, Malizos KN, Arvanitis DL, Zibis AH (2018). Anatomy learning from prosected cadaveric specimens versus three-dimensional software: a comparative study of upper limb anatomy. Ann Anat.

[13] Fruhstorfer BH, Palmer J, Brydges S, Abrahams PH (2011). The use of plastinated prosections for teaching anatomy—the view of medical students on the value of this learning resource. Clin Anat.

[14] Poh MZ, Swenson NC, Picard RW (2010). A wearable sensor for unobtrusive, long-term assessment of electrodermal activity. IEEE Trans Biomed Eng.

[15] Yammine K, Violato C (2016). The effectiveness of physical models in teaching anatomy: a meta-analysis of comparative studies. Adv in Health Sci Educ.

[16] Choi-Lundberg DL, Low TF, Patman P, Turner P, Sinha SN (2016). Medical student preferences for self-directed study resources in gross anatomy. Anat Sci Educ.

[17] Kassab S, Abu-Hijleh MF, Al-Shboul Q, Hamdy H (2005). Student-led tutorials in problem-based learning: educational outcomes and students’ perceptions. Med Teach.

[18] Krych AJ, March CN, Bryan RE, Peake BJ, Pawlina W, Carmichael SW (2005). Reciprocal peer teaching: students teaching students in the gross anatomy laboratory. Clin Anat.

[19] Evans DJR, Cuffe T (2009). Near-peer teaching in anatomy: an approach for deeper learning. Anat Sci Educ.

[20] Naqi SA (2014). Peer assisted learning as a formal instructional tool. J Coll Physicians Surg Pak.

[21] Iqbal H. Anatomy peer teaching in medical school: a literature review. MedEdPublish 5. 2020.

[22] Vagg T, Balta JY, Bolger A, Lone M. Multimedia in education: What do the students think?. Health Professions Education. 2020. 13 Jun 2020.

[23] Reidenberg JS, Laitman JT (2002). The new face of gross anatomy. Anat Rec.

[24] Sugand K, Abrahams P, Khurana A (2010). The anatomy of anatomy: a review for its modernization. Anat Sci Educ.

[25] Yeung JC, Fung K, Wilson TD (2011). Development of a computer-assisted cranial nerve simulation from the visible human dataset. Anat Sci Educ.

[26] Saxena V, Natarajan P, O’Sullivan PS, Jain S (2008). Effect of the use of instructional anatomy videos on student performance. Anat Sci Educ.

[27] Ma M, Fallavollita P, Seelbach I, Von Der Heide AM, Euler E, Waschke J, Navab N (2016). Personalized augmented reality for anatomy education. Clin Anat.

[28] Codd AM, Choudhury B (2011). Virtual reality anatomy: is it comparable with traditional methods in the teaching of human forearm musculoskeletal anatomy?. Anat Sci Educ.

[29] Park S, Kim Y, Park S, Shin JA (2019). The impacts of three-dimensional anatomical atlas on learning anatomy. Anat Cell Biol.

[30] Craik FI, Tulving E (1975). Depth of processing and the retention of words in episodic memory. J Exp Psychol Gen.

[31] Kerby J, Shukur ZN, Shalhoub J (2011). The relationships between learning outcomes and methods of teaching anatomy as perceived by medical students. Clin Anat.

[32] Bandyopadhyay R, Biswas R. Students’ perception and attitude on methods of anatomy teaching in a medical college of West Bengal, India. J Clin Diagn Res. 2017;11:AC10–AC14. 10.7860/JCDR/2017/26112.10666 PMC5713711 29207689

[33] Tayyem R, Qandeel H, Qsous G, Badran D, Bani-Hani K (2019). Medical students perception of current undergraduate anatomy teaching. Int J Morphol.

[34] Acland RD. *Acland’s video atlas of human anatomy*. Wolters Kluwer Health/Lippincott, Williams & Wilkins, Baltimore, MD. 2013. https://aclandanatomy.com/ [accessed 13 Feb 2018]

[35] Ozer MA, Govsa F, Bati AH (2017). Web-based teaching video packages on anatomical education. Surg Radiol Anat.

[36] Collins JP. Modern approaches to teaching and learning anatomy. BMJ. 2008;337. 10.1136/bmj.a1310 18782839

[37] Latorre RM, García-Sanz MP, Moreno M, Hernández F, Gil F, López O, Ayala MD, Ramirez G, Vazquez JM, Arencibia A, Henry RW (2007). How useful is plastination in learning anatomy?. J Vet Med Educ.

[38] Cahill DRLR (1997). The role of computers and dissection in teaching anatomy: a comment. Clin Anat.

[39] Agius A, Calleja N, Camenzuli C, Sultana R, Pullicino R, Zammit C, Calleja Agius J, Pomara C (2018). Perceptions of first-year medical students towards learning anatomy using cadaveric specimens through peer teaching. Anat Sci Educ.

