# Supplementary material for: Medical Students’ Opinions of Anatomy Teaching Resources and Their Role in Achieving Learning Outcomes
Source: Med Sci Educ. 2021 Oct 15;31(6):1903–10. doi: 10.1007/s40670-021-01436-2 (PMC8651893; doi:10.1007/s40670-021-01436-2)
Supplement: Supplementary file 1 — Supplementary file1 (PDF 61 kb) [file 40670_2021_1436_MOESM1_ESM.pdf]

Anatomy Teaching Resources and their Role in Achieving Learning Outcomes

I: Personal information:

Please provide the following demographic information.

1. Gender:

☐ Male ☐ Female ☐ Prefer not to specify

2. Age

☐ 20– 23 yrs ☐ 24 – 26 yrs

☐ 27 – 30 yrs ☐ Over 30 yrs

3. Please state your country of origin:

4. Previous Degree:

☐ Science Bachelor ☐ Non- Science Bachelor

☐ Science Master's ☐ Non- Science Master's

☐ Other, please specify_______________________

II: Anatomy Learning

5. Rank the following anatomy teaching aids from one to six with 1 (one) being most-preferred and 6 (six) being least preferred based on your learning experience.

Cadaveric prosections	
Dissection videos	
Printed resources (e.g. books)	
Electronic resources	
Plastic models	
Plastinated specimens	

6. Rate how the resources available in the anatomy lab helped you achieve the following learning outcomes by entering a number from zero to five (where zero is not helpful and five is most helpful).

	Cadaveric prosections	Dissection videos	Printed resources	Electronic resources	Plastic models	Plastinated specimens
Demonstrate an understanding of the gross anatomy						
Associate the various body systems and their synergic relationships						
Visualize the 3D anatomy of the structures						
Identify the different anatomical features of each structure						
Recall anatomical information						
Establish spatial relationship of different anatomical structures						
Appreciate the anatomical variations in the human body						
Understand and appreciate the clinical aspects of anatomy						
Identify and appreciate functional anatomy						
Recognize and explain the interrelationships within and between various anatomical structures						
Locate the various anatomical structures						
Develop a vocabulary of anatomical terminology to effectively communicate anatomical information						

Thank you for participating
